# Aging acceleration, not chronological age, is associated with cognitive performance in older adults: A cross-sectional study on the protective role of physical activity

**DOI:** 10.1097/MD.0000000000043431

**Published:** 2025-07-18

**Authors:** Yangyang Sun, Wei Wu, Xiaoqin Sun, Ling Ren, Zhengyun Ren, Gu Gong

**Affiliations:** a Department of Anesthesiology, The Affiliated Hospital, Southwest Medical University, Luzhou, China; b Department of Anesthesiology, The General Hospital of Western Theater Command, Chengdu, China; c College of Medicine, Southwest Jiaotong University, Chengdu, Sichuan, China.

**Keywords:** aging acceleration, cognitive performance, NHANES, PhenoAge, PhenoAgeAccel, physical activity

## Abstract

Cognitive decline in older adults is a growing public health concern, and traditional measures such as chronological age are insufficient for accurately assessing cognitive function. Phenotypic age (PhenoAge) and phenotypic age acceleration (PhenoAgeAccel), which reflect biological age and aging acceleration, may be better predictors of cognitive decline. Additionally, physical activity (PA) has been recognized for its protective effects on aging and cognitive health. This study explored the role of PhenoAge and PhenoAgeAccel in cognitive performance and investigated whether PA moderates this relationship. We used data from the National Health and Nutrition Examination Survey, which analyzed 1298 participants aged 60 years and older. PhenoAge was calculated using 10 biomarkers, and PhenoAgeAccel was derived as the difference between chronological age and PhenoAge. Cognitive performance was assessed using the Digit Symbol Substitution Test. The relationship between PhenoAge, PhenoAgeAccel, and low cognitive performance was analyzed using weighted logistic regression models. Subgroup and sensitivity analyses were conducted, and the interactions between PhenoAgeAccel and PA were evaluated. Both PhenoAge and PhenoAgeAccel scores were significantly associated with low cognitive performance. The highest quartiles of PhenoAge (odds ratio = 3.22, *P* = .025) and PhenoAgeAccel (odds ratio = 2.31, *P* = .022) were associated with higher odds of low cognitive performance. By contrast, chronological age did not show a significant relationship with cognitive performance. PA was found to moderate the association between PhenoAgeAccel and cognitive performance (*P* for interaction = .045). Higher levels of PA attenuated the impact of PhenoAgeAccel on cognitive decline. Receiver operating characteristic curve analysis showed that PhenoAge (area under the curve [AUC] = 0.562), PhenoAgeAccel (AUC = 0.589), and chronological age (AUC = 0.513) were significantly different. In conclusion, PhenoAgeAccel and PA are significant predictors of cognitive decline, with PA offering a protective effect against the impact of accelerated aging on cognition.

## 1. Introduction

With the acceleration of global population aging, cognitive impairment has become a significant public health issue, affecting the quality of life of older adults and imposing a considerable socioeconomic burden.^[1]^ Cognitive function encompasses multiple domains, including memory, executive function, language, and processing speed. Its decline not only compromises individuals’ ability to live independently but also markedly increases the demand for caregiving and medical resources.^[2]^ In recent years, studies based on large epidemiological databases, such as the National Health and Nutrition Examination Survey (NHANES), have consistently revealed that cognitive function in the elderly is influenced by a variety of physiological, metabolic, and lifestyle-related factors.^[3–5]^

Traditionally, chronological age has been widely used to assess the degree of aging and the related health risks in individuals.^[6]^ However, an increasing body of evidence suggests that chronological age alone fails to capture heterogeneity in aging rates and health status among individuals.^[7]^ Some people of the same age may exhibit markedly different physiological conditions and disease risk. Therefore, Levine and colleagues, using data from large US population studies such as the NHANES, proposed a novel biological age indicator known as phenotypic age (PhenoAge).^[8,9]^ PhenoAge integrates multiple clinical, physiological, and biochemical parameters (such as white blood cell counts, hemoglobin, blood glucose, C-reactive protein, etc), providing a more comprehensive reflection of an individual’s multisystem health status and aging process.^[10]^ The derived measure, phenotypic age acceleration (PhenoAgeAccel), represents the difference between an individual’s PhenoAge and chronological age and is used to quantify the rate of aging.^[11]^ Compared with chronological age or single biomarkers, PhenoAge and PhenoAgeAccel exhibit greater sensitivity and accuracy in predicting all-cause mortality, the onset of chronic diseases, and functional decline and are thus widely applied in epidemiological and mechanistic studies related to aging.^[12–16]^

Recent clinical studies have shown that accelerated aging (e.g., DunedinPoAm38) is significantly associated with motor cognitive risk syndromes.^[17]^ Other research has reported that biological aging is associated with increased monocyte inflammatory activity in older adults.^[18]^ Studies in population-based samples, such as NHANES, have further demonstrated that PhenoAgeAccel is closely related to mechanisms including inflammation, oxidative stress, and metabolic dysregulation, all of which are important biological pathways underlying cognitive decline.^[19,20]^ Meanwhile, lifestyle factors—particularly physical activity (PA)—have been widely recognized for their dual protective effects on biological aging and cognitive function.^[21]^ Regular PA not only helps slow biological aging but also significantly reduces the risk of cognitive decline through mechanisms such as improving metabolic status, lowering inflammation, and promoting neuroplasticity.^[22–24]^

To date, the relationship between accelerated aging and cognitive function in older adults has not been extensively studied. In addition, systematic studies exploring the complex interrelationships between accelerated aging, PA, and cognitive function in the elderly remain limited. Therefore, this study aimed to utilize the NHANES database to systematically examine the impact of accelerated aging, rather than chronological age, on cognitive function in older adults and to further explore the potential protective role of PA in this context. Elucidating these relationships is of great significance for accurately identifying high-risk populations for cognitive decline and for developing scientific interventions to promote healthy aging.

## 2. Methods

### 2.1. Study design and participants

This cross-sectional study used data from the NHANES, a comprehensive survey conducted by the National Center for Health Statistics (NCHS).

**Figure 1.**
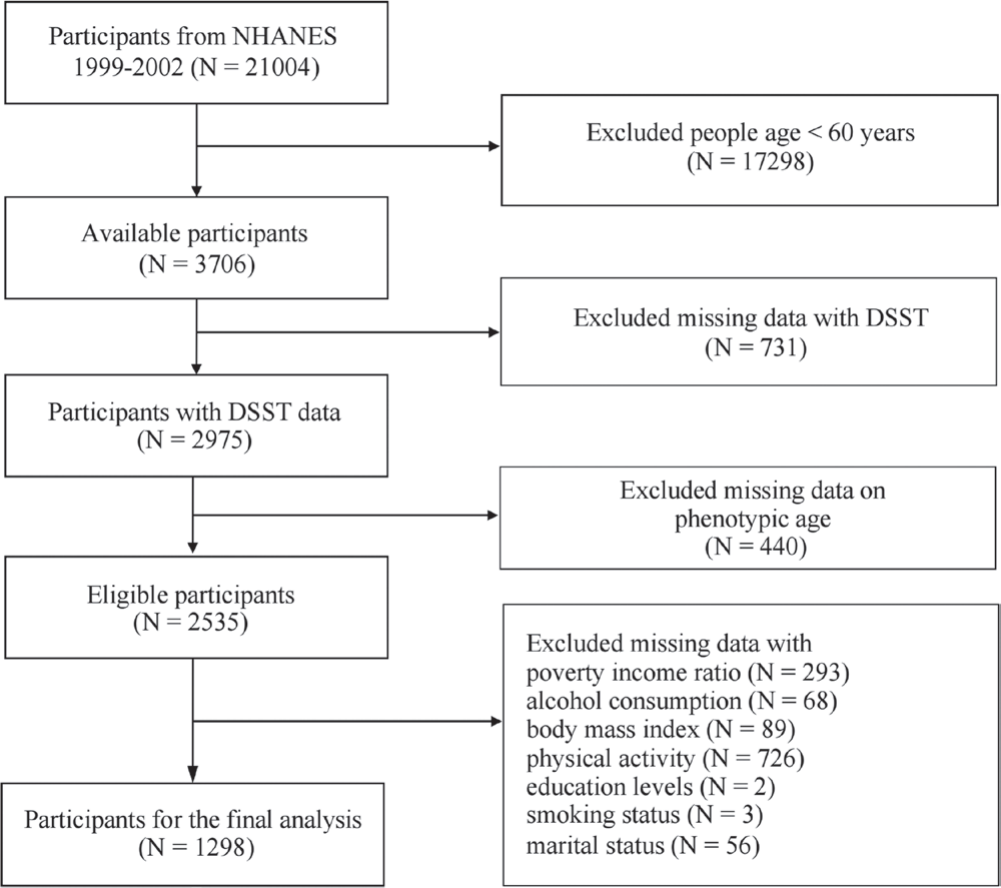
Flow chart of procedures for participants’ selection and inclusion. DSST = Digit Symbol Substitution Test, NHANES = National Health and Nutrition Examination Survey.

The analysis incorporated data from the cycles spanning 1999 to 2002, selected for the following reasons: the complete data necessary for calculating phenotypic age were only available from the 1999 to 2009 cycle and cognitive performance assessment was conducted only in the 1999 to 2002 and 2011 to 2014 cycles. Therefore, we focused on the intersection of these cycles to ensure the availability of both phenotypic age and cognitive performance data for all participants. Initially, the dataset included 21,004 participants; however, several inclusion and exclusion criteria were applied to ensure the relevance and reliability of the final sample. Participants aged under 60 years were excluded from the analysis because the cognitive performance assessment was only conducted in individuals aged 60 years and older. In addition, individuals with missing data on cognitive performance, phenotypic age, or other essential variables such as body mass index, PA level, and medical history (e.g., hypertension and diabetes) were also excluded. After these exclusions, the final sample comprised 1298 participants. A flowchart illustrating the inclusion and exclusion steps is presented in Figure 1, with specific details on the variables used for inclusion outlined in the figure. Survey weights were applied to the final sample to adjust for the complex survey design and provide a nationally representative estimate of the US adult population. The final study population corresponded to an estimated 19,679,400 weighted individuals.

### 2.2. Ethics statement

This study was conducted under the guidance of the NCHS and approved by the NCHS Institutional Review Board. Informed consent was obtained from all eligible participants before data collection and health assessment.

### 2.3. Definitions of phenotypic age and phenotypic age acceleration

PhenoAge has emerged as a more reliable predictor of health-related outcomes than chronological age alone. In this study, we defined PhenoAge as proposed by Levine et al^[25]^ and calculated it using 10 key aging-related variables: chronological age, creatinine (kidney function), albumin (liver function), glucose (metabolic health), lymphocyte percentage (immune function), C-reactive protein (inflammation), mean cell volume (immune system indicator), alkaline phosphatase (liver function), red blood cell distribution width (immune system indicator), and white blood cell count (immune function). Blood samples were collected at the mobile examination center according to standardized protocols, and the samples were securely stored in a dedicated facility for analysis. Using these data, we applied the algorithm for PhenoAge calculation, as outlined in earlier studies.^[25,26]^

In addition to calculating phenotypic age, we assessed aging acceleration by calculating the difference between chronological age and phenotypic age to derive the PhenoAgeAccel measures. These residuals were obtained by regressing chronological age on PhenoAge and were used to quantify aging acceleration, with a larger positive value representing a greater aging acceleration.

### 2.4. Cognitive performance assessment

The Digit Symbol Substitution Test (DSST), a subtest of the Wechsler Adult Intelligence Scale (third edition), was used to assess cognitive performance in participants aged ≥60 years in the NHANES 1999 to 2002 cycles. The DSST evaluates various cognitive domains with a primary focus on sustained attention, visual-motor speed, processing speed, learning capacity, and working memory.^[27]^

In this test, participants were required to match symbols to numbers based on a key provided at the top of the exercise form. The number of correct symbol–number pairings completed within a 120-second period was counted, yielding a total cognitive score, with a maximum score of 133. A higher score indicates better cognitive performance, with more blocks completed correctly.

Owing to the lack of a universally accepted cutoff for the DSST score to categorize low cognitive performance, we adopted the 25th percentile of the DSST score, representing the lowest quartile, as the threshold for low cognitive performance. This approach is consistent with the methods employed in existing literature.^[28,29]^ Furthermore, because age is a significant factor influencing cognitive performance, the DSST scores were further stratified by age groups: 60 to 69 years, 70 to 79 years, and ≥80 years. The cutoff values for low cognitive performance were set at 37 for the 60 to 69 age group, 33 for the 70 to 79 age group, and 29 for the ≥80 age group. Participants scoring below these cutoff values were categorized into the low cognitive performance group.

### 2.5. Definition of covariates

The collection and classification methods for key covariates such as hyperlipidemia, hypertension, and diabetes mellitus (DM) are outlined in Table S1, Supplemental Digital Content, https://links.lww.com/MD/P450. The definition of cardiovascular disease was based on previously established criteria that are commonly used in epidemiological research.^[30]^ PA was assessed using a standardized questionnaire that asked the participants to report their activities over the previous 30 days. The questionnaire recorded the type, frequency, and intensity of these activities, which were categorized into moderate- and vigorous-intensity levels based on their physiological impact.^[31,32]^ To calculate total physical activity volume, we summed the metabolic equivalent values associated with various activities related to work, recreation, and transportation. Further details of the methodology for defining and categorizing these covariates are available in the literature.^[33]^

### 2.6. Statistical analyses

Survey weights provided by the NHANES were applied to adjust for the complex survey design and ensure that the findings were representative of the US adult population. Descriptive statistics were used to summarize the characteristics of the study population. Continuous variables were expressed as means with standard deviations, while categorical variables were presented as frequencies and percentages. To compare differences between groups based on cognitive performance (low vs normal cognitive performance), chi-square tests were used for categorical variables, and analysis of variance was applied to continuous variables. Before performing regression analyses, collinearity diagnostics were conducted for all covariates, and variance inflation factors were calculated to assess potential multicollinearity. All variance inflation factor values were below 2.4, suggesting no significant collinearity.

We used weighted logistic regression models to explore the relationship between PhenoAge, PhenoAgeAccel, and low cognitive performance (measured using the DSST). The association was quantified using odds ratios (ORs) with 95% confidence intervals (CIs). To verify the assumptions of the logistic regression, the linearity between continuous independent variables and logit(p) transformation was examined. PhenoAge, PhenoAgeAccel, and total physical activity volume were categorized into quartiles (Q1–Q4), ranging from the lowest to highest levels, as outlined in Table S2, Supplemental Digital Content, https://links.lww.com/MD/P450. Subgroup analyses were performed to determine whether the association between PhenoAgeAccel and low cognitive performance differed across the various subgroups. Sensitivity analyses were conducted by running regressions without applying survey weights to evaluate the robustness of the results. Additionally, a histogram was used to visualize the distribution of PhenoAge and PhenoAgeAccel. To assess the potential nonlinear relationship between PhenoAge, PhenoAgeAccel, and cognitive performance, we employed restricted cubic spline analysis. Receiver operating characteristic curve analysis was performed to evaluate and compare the predictive accuracy of PhenoAge, PhenoAgeAccel, and chronological age in predicting cognitive performance. A *Z* test was used to assess whether the predictive performance of PhenoAge, PhenoAgeAccel, and chronological age differed significantly.

All statistical analyses were performed using R Studio (version 4.3.1, Posit, Boston) with the nhanesR package (version 0.9.4.3, Shanghai Zhishi Medical Technology Co., Ltd., Shanghai, China) following the STROBE (Strengthening the Reporting of Observational Studies in Epidemiology) guidelines for observational studies.

## 3. Results

### 3.1. Descriptive characteristics of the study population

The final analysis included 1298 participants, all aged ≥60 years, drawn from the NHANES cycles between 1999 and 2002. The demographic and clinical characteristics of the study population are summarized in Table 1. The mean PhenoAge of participants was 64.16 ± 0.36 years. A significant difference was observed between the low and normal cognitive performance groups. The low cognitive performance group had a significantly higher mean PhenoAge of 67.27 ± 0.68 years compared with 63.61 ± 0.39 years in the normal cognitive performance group (*P* < .001). Similarly, PhenoAgeAccel scores were significantly higher in the low cognitive performance group (1.20 ± 0.53) than in the normal cognitive performance group (−0.78 ± 0.25, *P *= .006). Participants who were Non-Hispanic White had a poverty–income ratio >3.5, had above high school education, were married or living with a partner, did not have DM, and did not have cardiovascular disease had a significantly lower proportion of low cognitive performance than their counterparts.

**Table 1:** Descriptive characteristics of the study population.

Characteristic	Total (N = 1298)	Digit Symbol Substitution Test		*P* value*s*
		Normal cognitive performance (n = 987)	Low cognitive performance (n = 311)	
Phenotypic age	64.16 ± 0.36	63.61 ± 0.39	67.27 ± 0.68	**<.001**
Phenotypic age acceleration	−0.49 ± 0.21	−0.78 ± 0.25	1.20 ± 0.53	**.006**
PA total MET	1129.8 ± 67.2	1109.9 ± 76.0	1243.3 ± 198.0	.556
Sex, n (%)				.236
Female	572 (48.94)	450 (86.72)	122 (13.28)	
Male	726 (51.06)	537 (83.63)	189 (16.37)	
Age, n (%)				.084
60–69	653 (53.28)	497 (86.59)	156 (13.41)	
70–79	436 (35.65)	330 (84.85)	106 (15.15)	
≥80	209 (11.07)	160 (79.16)	49 (20.84)	
Race/ethnicity, n (%)				**<.001**
Non-Hispanic White	831 (85.88)	725 (88.65)	106 (11.35)	
Non-Hispanic Black	158 (5.18)	95 (60.57)	63 (39.43)	
Hispanic	279 (6.32)	144 (60.32)	135 (39.68)	
Other race	30 (2.63)	23 (78.55)	7 (21.45)	
PIR, n (%)				**<.001**
<1.3	266 (14.62)	118 (57.38)	148 (42.62)	
1.3–3.5	561 (43.25)	436 (85.27)	125 (14.73)	
>3.5	471 (42.13)	433 (94.65)	38 (5.35)	
Education levels, n (%)				**<.001**
Below high school	213 (8.71)	62 (43.93)	151 (56.07)	
High school	514 (41.75)	389 (82.48)	125 (17.52)	
Above high school	571 (49.54)	536 (94.64)	35 (5.36)	
Marital status, n (%)				**.003**
Married/living with partner	890 (70.55)	696 (87.76)	194 (12.24)	
Never married	34 (2.65)	20 (66.89)	14 (33.11)	
Widowed/divorced/separated	374 (26.80)	271 (80.07)	103 (19.93)	
BMI, n (%)				.949
Underweight/normal	371 (30.26)	287 (85.08)	84 (14.92)	
Overweight	550 (40.71)	416 (85.51)	134 (14.49)	
Obese	377 (29.02)	284 (84.70)	93 (15.30)	
Smoking status, n (%)				.298
Never	581 (45.43)	439 (85.78)	142 (14.22)	
Former	574 (43.47)	449 (86.08)	125 (13.92)	
Now	143 (11.09)	99 (78.91)	44 (21.09)	
Alcohol consumption, n (%)				**.024**
Never	202 (15.95)	142 (80.64)	60 (19.36)	
Former	333 (24.44)	232 (80.18)	101 (19.82)	
Mild	529 (42.56)	439 (89.22)	90 (10.78)	
Moderate	142 (11.93)	110 (88.01)	32 (11.99)	
Heavy	92 (5.13)	64 (82.35)	28 (17.65)	
Hyperlipidemia, n (%)				.150
No	201 (14.33)	151 (80.63)	50 (19.37)	
Yes	1097 (85.67)	836 (85.90)	261 (14.10)	
Hypertension, n (%)				.094
No	431 (34.31)	346 (87.55)	85 (12.45)	
Yes	867 (65.69)	641 (83.89)	226 (16.11)	
Diabetes mellitus, n (%)				**.041**
No	944 (75.47)	746 (87.35)	198 (12.65)	
Prediabetes	93 (7.06)	69 (79.60)	24 (20.40)	
DM	261 (17.46)	172 (77.88)	89 (22.12)	
CVD, n (%)				**<.001**
No	1030 (79.56)	801 (87.06)	229 (12.94)	
Yes	268 (20.44)	186 (77.67)	82 (22.33)	

Bold values indicate statistical significance (*P* < .05).

BMI = body mass index, CVD = cardiovascular disease, DM = diabetes mellitus, MET = metabolic equivalent, PA = physical activity, PIR = poverty–income ratio.

### 3.2. Association between PhenoAge, PhenoAgeAccel, and low cognitive performance

The relationship between PhenoAge, PhenoAgeAccel, and low cognitive performance, as measured by DSST, was assessed using weighted logistic regression models. In all models, both PhenoAge and PhenoAgeAccel were significantly associated with low cognitive performance. Specifically, in the fully adjusted model, participants in the highest quartile of PhenoAge (Q4) had significantly higher odds of low cognitive performance than those in the lowest quartile (Q1). The OR for the Q4 group was 3.22 (95% CI: 1.17–8.85), with a *P* value of .025. Similarly, PhenoAgeAccel was significantly associated with low cognitive performance, with participants in the highest quartile (Q4) showing 2.31 times greater odds of low cognitive performance than those in the lowest quartile, with an OR of 2.31 (95% CI: 1.14–4.70) and *P* = .022 (Table 2). In contrast, chronological age was not significantly associated with low cognitive performance in the fully adjusted model (Table S3, Supplemental Digital Content, https://links.lww.com/MD/P450), suggesting that PhenoAge and PhenoAgeAccel may be more robust predictors of cognitive decline in older adults than chronological age.

**Table 2:** Adjusted association of phenotypic age (PhenoAge) and phenotypic age acceleration (PhenoAgeAccel) with low cognitive performance (Digit Symbol Substitution Test).

Exposure	Unadjusted model	Adjust 1	Adjust 2
	Odds ratio (95% CI) associated with low cognitive performance; *P* value		
PhenoAge			
Continuous	1.03 (1.01–1.05); **<.001**	1.04 (1.01–1.06); **.013**	1.03 (0.97–1.08); .191
Quartiles			
Q1	1 (Ref)	1 (Ref)	1 (Ref)
Q2	2.32 (1.17–4.61); **.018**	2.85 (1.18–6.86); **.022**	2.33 (1.07–5.05); **.033**
Q3	2.09 (0.90–4.85); .085	2.51 (0.78–8.11); .117	1.70 (0.54–5.33); .348
Q4	3.35 (1.97–5.71); **<.001**	4.80 (1.67–13.8); **.006**	3.22 (1.17–8.85); **.025**
*P* for trend	**<.001**	**.008**	.190
PhenoAgeAccel			
Continuous	1.03 (1.01–1.06); **.019**	1.03 (1.00–1.05); .040	1.02 (0.95–1.08); .397
Quartiles			
Q1	1 (Ref)	1 (Ref)	1 (Ref)
Q2	1.28 (0.69–2.39); .417	1.20 (0.64–2.23); .551	1.32 (0.68–2.58); .397
Q3	1.97 (1.07–3.63); **.032**	1.92 (1.05–3.51); **.037**	1.69 (0.90–3.19); .098
Q4	3.16 (1.63–6.14); **.001**	2.94 (1.54–5.62); **.002**	2.31 (1.14–4.70); **.022**
*P* for trend	**<.001**	**<.001**	.123

Unadjusted model: nonadjusted model. Adjust 1: adjust for age, sex, and race. Adjust 2: adjust for age, sex, race, body mass index, poverty–income ratio, education levels, marital status, smoking status, alcohol consumption, total physical activity volume, hyperlipidemia, hypertension, diabetes mellitus, and cardiovascular disease. Bold values indicate statistical significance (*P* < .05).

### 3.3. Subgroup analyses and sensitivity analysis

**Figure 2.**
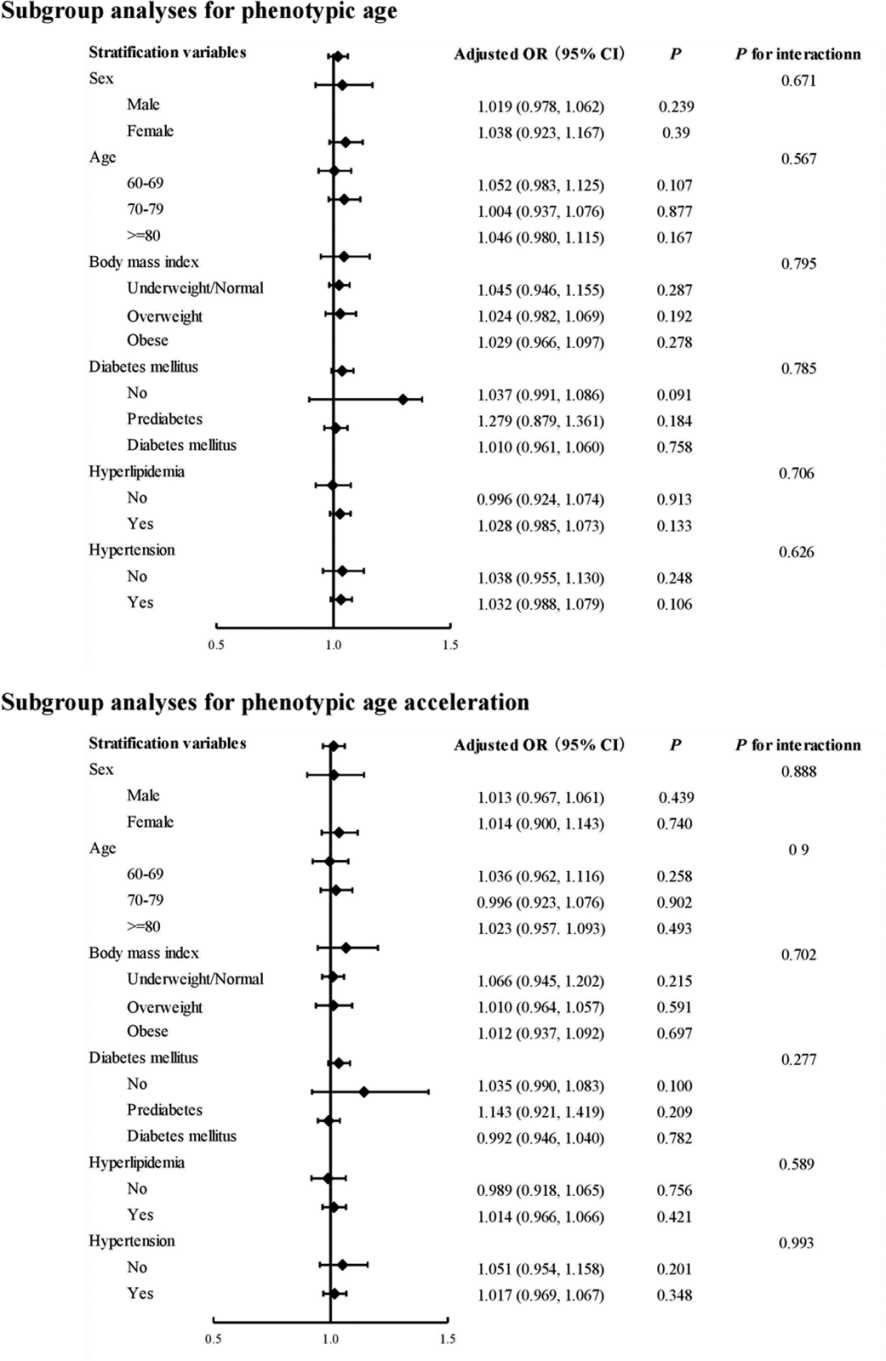
The odds ratio (OR) of low cognitive performance was examined in participants with either phenotypic age or phenotypic age acceleration, performing subgroup analysis. All analyses were adjusted for age, sex, race, body mass index, poverty–income ratio, education levels, marital status, smoking status, alcohol consumption, metabolic equivalent, hyperlipidemia, hypertension, diabetes mellitus, and cardiovascular disease, but not for the specific stratification variables of interest. CI == confidence interval.

The results of the subgroup analyses are shown in Figure 2. Overall, the association between PhenoAge, PhenoAgeAccel, and low cognitive performance remained consistent across all the subgroups. The *P* values for interaction were all >.05, indicating that the relationship between aging measures and cognitive performance did not differ significantly across various subgroups, including age, sex, body mass index, DM, hyperlipidemia, and hypertension categories. To test the robustness of the findings, sensitivity analyses were performed using an alternative model that did not apply survey weights (Table 3). The results from these analyses were largely consistent with those of the primary analyses, confirming that higher PhenoAge and PhenoAgeAccel were significantly associated with higher odds of low cognitive performance across all models. This suggests that the findings are not significantly affected by the choice of statistical model or the application of survey weights.

**Table 3:** Adjusted association of phenotypic age (PhenoAge) and phenotypic age acceleration (PhenoAgeAccel) with low cognitive performance (Digit Symbol Substitution Test) without weighting in sensitivity analysis.

Exposure	Unadjusted model	Adjust 1	Adjust 2
	Odds ratio (95% CI) associated with low cognitive performance; *P* value		
PhenoAge			
Continuous	1.02 (1.01–1.03); **<.001**	1.03 (1.02–1.05); .**001**	1.03 (0.99–1.07); .089
Quartiles			
Q1	1 (Ref)	1 (Ref)	1 (Ref)
Q2	1.54 (1.06–2.24); .**026**	2.01 (1.30–3.11); .**003**	1.80 (1.16–2.79); .**010**
Q3	1.73 (1.14–2.61); .**012**	2.23 (1.20–4.12); .**014**	1.73 (0.90–3.34); .098
Q4	1.72 (1.26–2.34); .**001**	3.03 (1.88–4.90); **<.001**	2.46 (1.40–4.34); .**003**
*P* for trend	**<.001**	**<.001**	.103
PhenoAgeAccel			
Continuous	1.03 (1.01–1.05); .**005**	1.03 (1.01–1.05); .**007**	1.02 (0.98–1.07); .175
Quartiles			
Q1	1 (Ref)	1 (Ref)	1 (Ref)
Q2	1.16 (0.79–1.70); .442	1.14 (0.76–1.70); .512	1.28 (0.84–1.94); .239
Q3	1.62 (1.10–2.38); .**017**	1.60 (1.04–2.46); .**032**	1.69 (1.13–2.53); .**012**
Q4	2.31 (1.48–3.60); **<.001**	2.30 (1.51–3.51); **<.001**	2.20 (1.31–3.70); .**004**
*P* for trend	**<.001**	**<.001**	.078

Unadjusted model: nonadjusted model. Adjust 1: adjust for age, sex, and race. Adjust 2: adjust for age, sex, race, body mass index, poverty–income ratio, education levels, marital status, smoking status, alcohol consumption, total physical activity volume, hyperlipidemia, hypertension, diabetes mellitus, and cardiovascular disease. Bold values indicate statistical significance (*P* < .05).

In summary, both subgroup and sensitivity analyses consistently supported the harmful association between PhenoAge, PhenoAgeAccel, and low cognitive performance, further reinforcing the robustness of our results.

### 3.4. Regulation of PA on PhenoAgeAccel and cognitive performance

We did not find a significant interaction between PhenoAge and PA on the risk of low cognitive performance (*P* for interaction = .602, Table S4, Supplemental Digital Content, https://links.lww.com/MD/P450). However, a significant interaction was observed between PhenoAgeAccel and PA, suggesting that the relationship between PhenoAgeAccel and low cognitive performance was moderated by PA level (*P* for interaction = .045, Table 4). Specifically, higher PA levels were associated with a reduced risk of low cognitive performance, even as PhenoAgeAccel increased. This indicates that PA may have a protective effect, potentially reducing the harmful impact of accelerated aging on cognitive performance.

**Table 4:** Adjusted association of phenotypic age acceleration (PhenoAgeAccel) with low cognitive performance (Digit Symbol Substitution Test) in different total physical activity volume (PA total MET) subgroups.

Variables	Odds ratio (95% CI); *P* value*	*P* for interaction
PA total MET Q1		**.045**
PhenoAgeAccel Q1	1 (Ref)	
PhenoAgeAccel Q2	0.810 (0.305–2.151); 0.663	
PhenoAgeAccel Q3	2.641 (0.742–9.398); 0.128	
PhenoAgeAccel Q4	1.910 (0.462–7.902); 0.358	
PA total MET Q2		
PhenoAgeAccel Q1	1 (Ref)	
PhenoAgeAccel Q2	17.81 (5.813–54.60); **<.001**	
PhenoAgeAccel Q3	2.378 (0.515–10.99); 0.256	
PhenoAgeAccel Q4	13.81 (4.421–43.13); **<.001**	
PA total MET Q3		
PhenoAgeAccel Q1	1 (Ref)	
PhenoAgeAccel Q2	1.268 (0.256–6.280); 0.763	
PhenoAgeAccel Q3	1.895 (0.483–7.440); 0.346	
PhenoAgeAccel Q4	6.507 (1.252–33.82); **0.027**	
PA total MET Q4		
PhenoAgeAccel Q1	1 (Ref)	
PhenoAgeAccel Q2	0.628 (0.122–3.225); 0.565	
PhenoAgeAccel Q3	1.253 (0.343–4.573); 0.724	
PhenoAgeAccel Q4	1.170 (0.341–4.014); 0.796	

Bold values indicate statistical significance (*P* < .05).

* The analysis was adjusted for age, sex, race, body mass index, poverty–income ratio, education levels, marital status, smoking status, alcohol consumption, hyperlipidemia, hypertension, diabetes mellitus, and cardiovascular disease.

**Figure 3.**
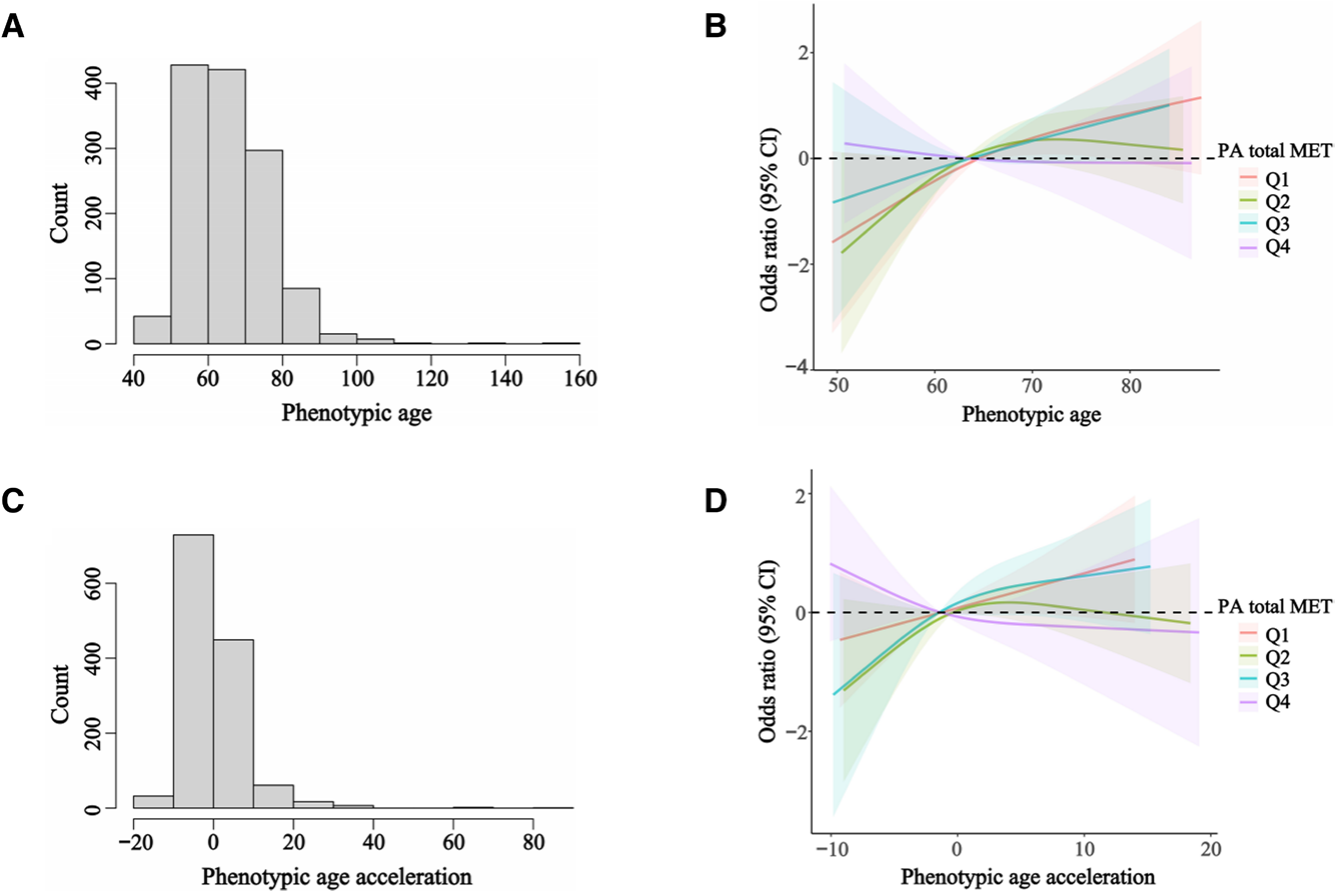
The distribution of phenotypic age (A) and phenotypic age acceleration (C). The full-adjusted relationship between phenotypic age and phenotypic age acceleration with low cognitive performance (Digit Symbol Substitution Test) using Restricted Cubic Spline (B and D). The solid line represents the fitted nonlinear curve. The area adjacent to the solid line represents the 95% confidence interval. All quartiles, except Q2, show a nonlinear *P* value >.05.

Figure 3 presents the distribution of both PhenoAge and PhenoAgeAccel, with both measures following an approximately normal distribution. Further exploration through restricted cubic spline analysis showed that the relationship between PhenoAge and PhenoAgeAccel and low cognitive performance was linear in most PA quartiles. All PA quartiles, except for Q2, showed nonlinear *P* values >.05. We found that both PhenoAge and PhenoAgeAccel were positively correlated with the incidence of low cognitive performance. Moreover, this relationship weakened with an increase in PA intensity, which was the weakest in the PA Q4 group.

In summary, the interaction between PhenoAgeAccel and PA underscores the importance of PA in modulating the effects of accelerated aging on cognitive decline. This highlights the potential of PA to counteract the negative effects of accelerated aging on cognition.

### 3.5. Predictive accuracy of PhenoAge, PhenoAgeAccel, and chronological age

**Figure 4.**
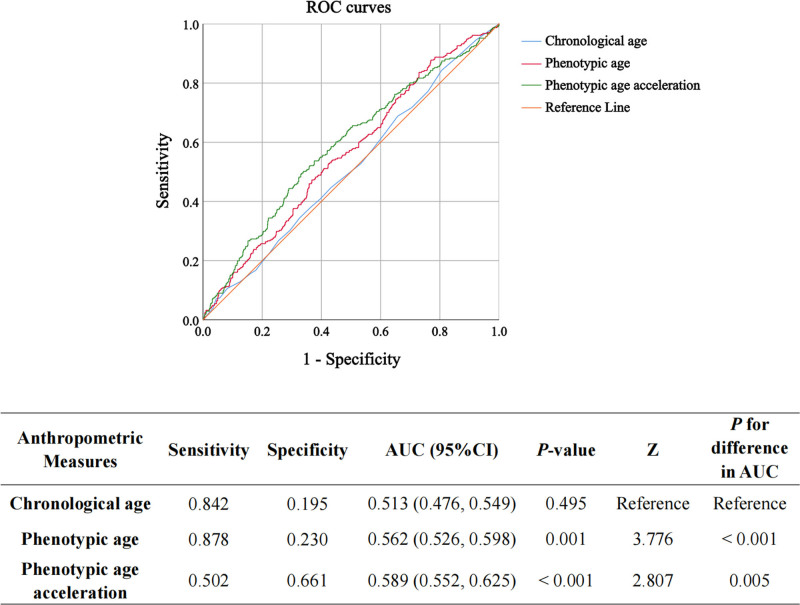
The receiver operating characteristic (ROC) curves for the prediction of low cognitive performance. The *Z* test of the area under the ROC curve (AUC) is used to assess the significant difference in predictive performance between the prediction models.

Receiver operating characteristic curve analysis was performed to assess the predictive accuracy of PhenoAge, PhenoAgeAccel, and chronological age in relation to cognitive performance. As shown in Figure 4, both PhenoAge and PhenoAgeAccel demonstrated a higher area under the curve (AUC) than chronological age. The AUC for PhenoAge was 0.562, for PhenoAgeAccel was 0.589, and 0.513 for chronological age. These results indicate that PhenoAge and PhenoAgeAccel are better predictors of low cognitive performance than chronological age. The *Z* test for differences in AUC confirmed that the difference in predictive performance between PhenoAge, PhenoAgeAccel, and chronological age was statistically significant (*P* < .001 for PhenoAge vs chronological age and *P* = .005 for PhenoAgeAccel vs chronological age). This further supports the conclusion that both PhenoAge and PhenoAgeAccel are more reliable predictors of cognitive decline than chronological age in older adults.

## 4. Discussion

In this study, we explored the relationship between PhenoAge, PhenoAgeAccel, and low cognitive performance in older adults using data from NHANES cycles from 1999 to 2002. Our findings suggest that PhenoAge and PhenoAgeAccel are more strongly associated with cognitive decline than chronological age and that PA may have a protective effect in modulating this relationship.

Although chronological age remains a commonly used measure in epidemiological studies, our findings highlight the limitations of relying on chronological age alone to assess cognitive function. Chronological age only represents the passage of time since birth and does not reflect the biological processes that affect an individual’s cognitive health. This is particularly important in older adults, as aging is not a uniform process: 2 individuals of the same chronological age may experience very different biological aging rates and cognitive outcomes.^[25,34]^ In contrast, PhenoAge and PhenoAgeAccel, which consider biological markers of aging, provide a more accurate measure of an individual’s physiological state and are better predictors of cognitive performance. PhenoAge integrates biomarkers related to organ function (e.g., liver and kidney), metabolic health (e.g., glucose), and inflammation, offering a holistic view of biological aging.^[25]^ This is crucial because these factors are known to influence brain health and are directly linked to cognitive decline. For instance, increased systemic inflammation, oxidative stress, and poor metabolic health have all been shown to accelerate cognitive aging, which chronological age alone does not capture.^[35–37]^ Furthermore, PhenoAgeAccel, which reflects the difference between PhenoAge and chronological age, provides an indication of the rate at which an individual is aging relative to their chronological age. Individuals with a high PhenoAgeAccel score are undergoing accelerated aging, which is strongly associated with cognitive impairment in our study.

One of the most compelling findings of this study was the moderating effect of PA on the relationship between PhenoAgeAccel and cognitive performance. Specifically, we observed that higher levels of PA attenuated the association between PhenoAgeAccel (aging acceleration) and low cognitive performance. This suggests that individuals who engage in higher PA levels may be able to mitigate the cognitive risks associated with accelerated aging. This result is consistent with existing literature that highlights the protective role of PA in reducing the negative effects of aging on cognitive function.^[38,39]^ Our findings suggest that PA may offer a potential intervention to help prevent or delay the onset of cognitive decline in aging populations, particularly for those showing signs of accelerated aging as indicated by higher PhenoAgeAccel. These results underscore the importance of maintaining an active lifestyle, not only to promote overall health but also to protect cognitive function as we age. In line with prior research, it is likely that PA influences brain health through multiple mechanisms, including the promotion of neuroplasticity, the ability of the brain to form new neural connections, and the enhancement of cerebral blood flow, which improves the delivery of oxygen and nutrients to the brain tissues.^[40–42]^ Furthermore, PA has been shown to reduce inflammation, regulate metabolism, and improve cardiovascular health, all of which are beneficial in maintaining cognitive function and preventing cognitive decline.^[43,44]^

While our study offers important insights into the relationship between aging acceleration, PA, and cognitive performance, it is not without its limitations. The cross-sectional design limits our ability to draw causal conclusions. Future longitudinal studies are needed to assess the directionality of the relationships observed in this study. Additionally, while we controlled for various confounding factors, there may still be residual confounding factors that could have affected the findings. Further research should explore the mechanisms through which PhenoAgeAccel influences cognitive decline and the potential benefits of PA in mitigating these effects. Additionally, the use of self-reported PA in the NHANES may introduce bias due to recall errors, and future studies should consider using objective measures of PA to improve the accuracy of the data.

## 5. Conclusions

This study highlights the importance of PhenoAge and PhenoAgeAccel as predictors of low cognitive performance in older adults and underscores the protective role of PA in mitigating the effects of accelerated aging. Our findings suggest that interventions aimed at reducing aging acceleration, particularly through PA, could be key strategies for preventing or delaying cognitive decline in older populations. Future research should focus on exploring the underlying mechanisms linking aging acceleration and cognitive decline, as well as evaluating the long-term benefits of PA interventions on cognitive health.

## Acknowledgments

We sincerely appreciate the hard work and dedication of the NHANES staff and the principal investigators. We are especially grateful to the study participants, whose valuable contributions have been essential for furthering scientific research and public health insights.

## Author contributions

**Conceptualization:** Yangyang Sun, Gu Gong.

**Investigation:** Yangyang Sun, Gu Gong.

**Writing – original draft:** Yangyang Sun.

**Writing – review & editing:** Yangyang Sun, Wei Wu, Xiaoqin Sun, Ling Ren, Zhengyun Ren, Gu Gong.

**Methodology:** Wei Wu, Xiaoqin Sun, Ling Ren, Zhengyun Ren, Gu Gong.

**Data curation:** Zhengyun Ren.

**Formal analysis:** Zhengyun Ren.

## Supplementary Material


