# Supplementary material for: Aging acceleration, not chronological age, is associated with cognitive performance in older adults: A cross-sectional study on the protective role of physical activity
Source: Medicine (Baltimore). 2025 Jul 18;104(29):e43431. doi: 10.1097/MD.0000000000043431 (PMC12282797; doi:10.1097/MD.0000000000043431)
Supplement: Supplementary file 1 [file medi-104-e43431-s001.pdf]

Table S1. The detailed overview of how we obtained information on hypertension, hyperlipidemia, and diabetes.

**Hypertension**	The inclusion of hypertension was determined by considering both the results of a questionnaire survey and three separate blood pressure measurements taken from the participants. The participants were asked the following questions: " {Have you/Has SP} ever been told by a doctor or other health professional that {you/s/he} had hypertension, also called high blood pressure? Because of {your/SP's} (high blood pressure/hypertension), {have you/has s/he} ever been told to . . . take prescribed medicine?" And Hypertension was also confirmed if the blood pressure measurements exceeded 140/90 mmHg.
**Hyperlipidemia**	The information regarding hyperlipidemia is primarily obtained based on the following criteria<br>1. High Triglycerides (TG): TG levels greater than or equal to 150 mg/dL.<br>2. High Total Cholesterol (TC): TC levels greater than or equal to 200 mg/dL [5.18 mmol/L].<br>3. Elevated Low-density lipoprotein (LDL)-Cholesterol: LDL cholesterol levels greater than or equal to 130 mg/dL [3.37 mmol/L].<br>4. Low high-density lipoprotein (HDL)-Cholesterol: In males, HDL cholesterol levels below 40 mg/dL [1.04 mmol/L]. In females, HDL cholesterol levels below 50 mg/dL [1.30 mmol/L].<br>5. The use of lipid-lowering medications.
**Diabetes mellitus**	The diagnostic criteria for diabetes are:<br>1. doctor told you have diabetes ({Other than during pregnancy, {have you/has SP}/ {Have you/Has SP}} ever been told by a doctor or health professional that {you have/{he/she/SP} has} diabetes or sugar diabetes?),<br>2. glycohemoglobin HbA1c (%) >= 6.5,<br>3. fasting glucose (mmol/l) >= 7.0,<br>4. random blood glucose (mmol/l) >= 11.1,<br>5. two-hour OGTT blood glucose (mmol/l) >= 11.1,<br>6. Use of diabetes medication or insulin,<br>Prediabetes: impaired fasting glycaemia and impaired glucose tolerance.

Table S2. Details of phenotypic age, phenotypic age acceleration and PA total MET division.

Variables	Division details	Corresponding range	Frequency	Percentage
**phenotypic age**	Q1	[42.246, 57.169]	325	25.04%
	Q2	(57.169, 63.613]	324	24.96%
	Q3	(63.613, 72.216]	324	24.96%
	Q4	(72.216, 156.58]	325	25.04%
**phenotypic age acceleration**	Q1	[-14.277, -4.461]	325	25.04%
	Q2	(-4.461, -1.188]	324	24.96%
	Q3	(-1.188, 2.637]	324	24.96%
	Q4	(2.637, 84.006]	325	25.04%
**PA total MET**	Q1	[0.933, 189]	347	26.73%
	Q2	(189, 504]	303	23.34%
	Q3	(504, 1310.908]	323	24.88%
	Q4	(1310.908, 20160]	325	25.05%

Table S3. Adjusted association of phenotypic age (PhenoAge), phenotypic age acceleration (PhenoAgeAccel) and chronological age with Digit Symbol Substitution Test (DSST).

Exposure	Unadjusted model	Adjust 1	Adjust 2
	Odds ratio (95% CI) associated with low cognitive performance		
**PhenoAge**			
Q1	1 (Ref)	1 (Ref)	1 (Ref)
Q2	2.32 (1.17, 4.61); **0.018**	2.85 (1.18, 6.86); **0.022**	2.33 (1.07, 5.05); **0.033**
Q3	2.09 (0.90, 4.85); 0.085	2.51 (0.78, 8.11); 0.117	1.70 (0.54, 5.33); 0.348
Q4	3.35 (1.97, 5.71); < **0.001**	4.80 (1.67, 13.8); **0.006**	3.22 (1.17, 8.85); **0.025**
**Chronological age**			
60-69		1 (Ref)	1 (Ref)
70-79		0.77 (0.43, 1.39); 0.369	0.58 (0.33, 1.01); 0.050
>=80		0.94 (0.37, 2.40); 0.887	0.83 (0.31, 2.20); 0.696
**PhenoAgeAccel**			
Q1	1 (Ref)	1 (Ref)	1 (Ref)
Q2	1.28 (0.69, 2.39); 0.417	1.20 (0.64, 2.23); 0.551	1.32 (0.68, 2.58); 0.397
Q3	1.97 (1.07, 3.63); **0.032**	1.92 (1.05, 3.51); **0.037**	1.69 (0.90, 3.19); 0.098
Q4	3.16 (1.63, 6.14); **0.001**	2.94 (1.54, 5.62); **0.002**	2.31 (1.14, 4.70); **0.022**
**Chronological age**			
60-69		1 (Ref)	1 (Ref)
70-79		1.24 (0.87, 1.77); 0.211	0.77 (0.49, 1.19); 0.224
>=80		2.10 (1.26, 3.49); **0.007**	1.44 (0.78, 2.65); 0.230

Unadjusted model: non-adjusted model.

Adjust 1: Adjust for age, sex, race.

Adjust 2: Adjust for age, sex, race, body mass index, poverty income ratio, education levels, marital status, smoking status, alcohol consumption, PA total MET, hyperlipidemia, hypertension, diabetes mellitus and cardiovascular disease.

Table S4. Adjusted association of phenotypic age (PhenoAge) with low cognitive performance (Digit Symbol Substitution Test) in different PA total MET subgroups.

**Variables**	**Odds ratio (95% CI); *P*-value***	***P* for interaction**
**PA total MET Q1**		0.602
PhenoAge Q1	1 (Ref)	
PhenoAge Q2	1.974 (0.571, 6.821); 0.271	
PhenoAge Q3	2.257 (0.457, 11.13); 0.305	
PhenoAge Q4	10.74 (1.146, 100.7); **0.038**	
**PA total MET Q2**		
PhenoAge Q1	1 (Ref)	
PhenoAge Q2	1.924 (0.544, 6.812); 0.297	
PhenoAge Q3	3.726 (1.033, 13.44); **0.045**	
PhenoAge Q4	2.699 (0.536, 13.58); 0.218	
**PA total MET Q3**		
PhenoAge Q1	1 (Ref)	
PhenoAge Q2	2.441 (0.528, 11.28); 0.242	
PhenoAge Q3	2.646 (0.513, 13.64); 0.234	
PhenoAge Q4	5.952 (0.622, 56.93); 0.117	
**PA total MET Q4**		
PhenoAge Q1	1 (Ref)	
PhenoAge Q2	2.158 (0.850, 5.478); 0.102	
PhenoAge Q3	0.468 (0.113, 1.939); 0.283	
PhenoAge Q4	0.591 (0.191, 1.828); 0.348	

* The analyse was adjusted for age, sex, race, body mass index, poverty income ratio, education levels, marital status, smoking status, alcohol consumption, hyperlipidemia, hypertension, diabetes mellitus and cardiovascular disease.
